# Tachykinins, new players in the control of reproduction and food intake: A comparative review in mammals and teleosts

**DOI:** 10.3389/fendo.2022.1056939

**Published:** 2022-12-16

**Authors:** Aurora Campo, Sylvie Dufour, Karine Rousseau

**Affiliations:** ^1^ Muséum National d’Histoire Naturelle, Research Unit Unité Mixte de Recherche Biologie des Organsimes et Ecosystèmes Aquatiques (UMR BOREA), Biology of Aquatic Organisms and Ecosystems, Centre National pour la Recherche Scientifique (CNRS), Institut de Recherche pour le Développemen (IRD), Sorbonne Université, Paris, France; ^2^ Volcani Institute, Agricultural Research Organization, Rishon LeTsion, Israel; ^3^ Muséum National d’Histoire Naturelle, Research Unit PhyMA Physiologie Moléculaire et Adaptation CNRS, Paris, France

**Keywords:** tachykinins, neurokinins, substance P, endokinin/hemokinin, reproduction, food intake, mammals, teleosts

## Abstract

In vertebrates, the tachykinin system includes tachykinin genes, which encode one or two peptides each, and tachykinin receptors. The complexity of this system is reinforced by the massive conservation of gene duplicates after the whole-genome duplication events that occurred in vertebrates and furthermore in teleosts. Added to this, the expression of the tachykinin system is more widespread than first thought, being found beyond the brain and gut. The discovery of the co-expression of neurokinin B, encoded by the tachykinin 3 gene, and kisspeptin/dynorphin in neurons involved in the generation of GnRH pulse, in mammals, put a spotlight on the tachykinin system in vertebrate reproductive physiology. As food intake and reproduction are linked processes, and considering that hypothalamic hormones classically involved in the control of reproduction are reported to regulate also appetite and energy homeostasis, it is of interest to look at the potential involvement of tachykinins in these two major physiological functions. The purpose of this review is thus to provide first a general overview of the tachykinin system in mammals and teleosts, before giving a state of the art on the different levels of action of tachykinins in the control of reproduction and food intake. This work has been conducted with a comparative point of view, highlighting the major similarities and differences of tachykinin systems and actions between mammals and teleosts.

## 1 Introduction

Tachykinins (TAC) are members of a large family of peptides present from cnidaria [for reviews (1, 2)]: to bilateria [for reviews (1–8):. Tachykinins are usually considered as brain and gut peptides, as they are mainly expressed in neurons from the central nervous system and from the gastrointestinal tract. However, they are also present in non-neuronal cells, such as the immune and inflammatory cells of mammals, and various tissues like the skin of amphibians, as well as the salivary gland of mosquito and octopus, where they serve for exocrine secretion [for reviews (1, 4, 6, 8)]. In addition, in sea squirt, they are found in endostyle and gonad (8), where they act as neurotransmitters of endocrine and local autocrine/paracrine regulations [for reviews (4, 8)].

Since the discovery of the co-expression of neurokinin B, encoded by tachykinin 3 gene (*tac3*), and kisspeptin/dynorphin in neurons involved in the generation of GnRH pulses in mammals, rekindled attention has emerged for studying tachykinins in vertebrate reproductive physiology. Reproduction is classically controlled by the hypothalamus–pituitary–gonad (HPG) neuroendocrine axis in vertebrates [for review (9)]. The gonadotropin-releasing hormone (GnRH), produced and released by hypothalamic neurons, acts on the pituitary to stimulate the synthesis and release of gonadotropins, luteinizing hormone (LH), and follicle-stimulating hormone (FSH). These pituitary hormones act themselves on the gonads to control gametogenesis and the production of sex steroids, mainly estrogens in females and androgens in males. These peripheral hormones exert feedbacks at brain and pituitary levels to regulate GnRH and gonadotropin production. The hypothalamus is also the cerebral center involved in the control of food intake, integrating both external and internal factors and producing neuropeptides stimulating (orexigenic) or inhibiting (anorexigenic) appetite [for reviews (10–13)].

In vertebrates, feeding and reproduction are linked processes, as the presence of sufficient energy reserves is critical to achieve successful reproduction [for reviews (14, 15)]. Any state of negative energy balance thus affects not only central appetite-regulating systems but, often, also reproductive pathways and reproductive performance. Hypothalamic hormones classically involved in the control of reproduction, such as kisspeptin, are reported to regulate appetite and energy homeostasis as well, in mammals [for reviews (16–18)] and teleosts [for review (19)].

As teleosts represent the most diversified group of vertebrates, with nearly 30,000 species, species-specific regulating mechanisms are often encountered inside this lineage. In addition, some physiological differences exist between fish and mammalian regulatory mechanisms, even if major regulatory features are conserved. Some differences may be due to anatomical specificities of teleost neuroendocrine systems such as direct neuronal innervation and cell regionalization of the pituitary [for reviews (9, 20)]. Major breakthroughs in the studies of neuropeptide actions in teleosts have been allowed thanks to recently available published genomes and novel genome editing technics. These new tools are of particular interest and necessity in this group of vertebrates, as due to the teleost-specific whole-genome duplication (3R), teleosts possess an expanded number of genes encoding hormones/peptides that will share initial pleiotropic functions (subfunctionalization) or get new functions (neofunctionalization) [for review (9)].

With a comparative perspective, the purpose of this review is to provide a general overview of the tachykinins and their receptors in mammals and teleosts and then focus on the state-of-the-art literature on the different levels of action of tachykinins in the control of reproduction and food intake in these two groups of vertebrates.

## 2 Tachykinin system

Some of the first peptides of the tachykinin (TAC) family were discovered in neurons in mammals and therefore named neurokinins (NK). However, many subsequent data showed their production by non-neuronal cells. Especially the discovery in 2000 by Zhang and collaborators of a third tachykinin gene, PPT-C (21), renamed *tac4* and encoding several new tachykinin peptides, with widespread peripheral distributions and with a preferred receptor NK1 receptor, led to debates on their nomenclature [for reviews (22, 23)]. A revised nomenclature was proposed, with the preferred term “tachykinin” compared with “neurokinin,” which then appeared inappropriate. Similarly, for tachykinin receptors, for example, the NK1 receptor can no longer be defined only as a substance P (SP) receptor [for reviews (23, 24)]. More recently, the Human Genome Organization (HUGO) Gene Nomenclature Committee approved the names TACR1, TACR2, and TACR3 for the three TAC receptors (25). This nomenclature will be adopted in our review. In the following text, we will use TAC for tachykinin peptides and *tac* for tachykinin genes and transcripts, and likewise TACR and *tacr* for the receptors.

A recent review highlights the widespread distribution and the functional pleiotropy of TACs and their receptors with a special focus on invertebrates (2) and is complementary to our present comparative review in vertebrates.

### 2.1 Tachykinins

The evolutionary scenario of tachykinins in chordates suggests that an ancestral *tac* gene in proto-chordates generated four paralogs [(26), for reviews (1, 27)] after the two whole-genome duplication rounds (1R/2R) which occurred in early vertebrates (28, 29). A possible loss of one of the four paralogs occurred before the split of the ray-finned fish, actinopterygians (leading to teleost fish), and the lobe-finned fish, sarcopterygians (leading to mammals) [(26), for review (1)]. Among teleosts, a specific third-genome duplication (3R) produced a tetraploidization, followed by gene loss or conservation of duplicated paralogs (30, 31). Our recent study shows a wide conservation of the duplicated tachykinin genes in the teleost fish lineage (Campo et al. in preparation), thus increasing the scope of previous research (26).

**Figure 1:**
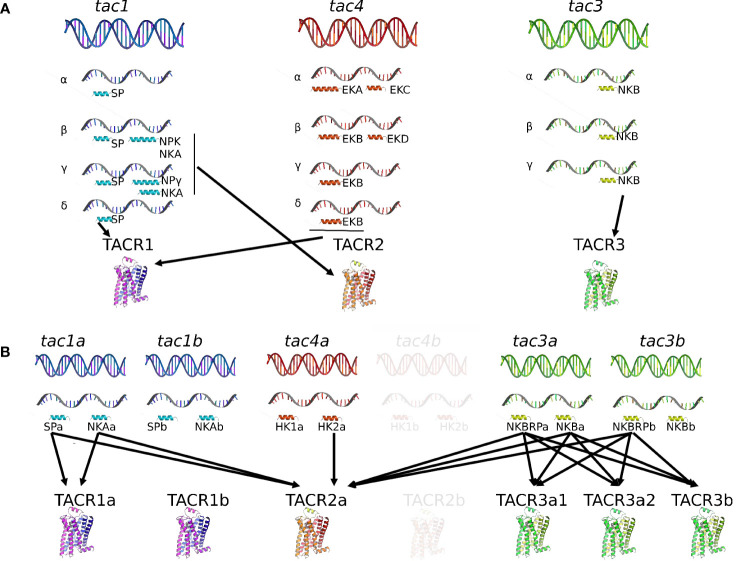
Comparison of the tachykinin system in a mammal, the human, and in a teleost, the grass carp. In human **(A)**, the tachykinin system comprises three *tac* genes (*tac1*, *tac2*, and *tac3*) encoding up to 10 different TAC peptides (SP, NKA, NPK, and NPγ for the *tac1* gene; HK1, EKA, EKB, EKC, and EKC for the *tac4* gene; NKB for the *tac3* gene) due to the existence of various spliced variants (α, β, γ, and δ for the tac1 and tac4 genes; (α, β, and γ for the tac3 gene). These human TAC peptides bind to three TACR (TACR1, TAC2, and TACR3) with different affinities: SP, HK1, EKA, and EKB for TACR1; NKA, NPK, and NPγ for TACR2; NKB for TACR3. In teleosts **(B)**, due to the teleost-specific whole-genome duplication (3R), duplicates for *tac1* (*tac1a* and *tac1b*), *tac3* (*tac3a* and *tac3b*), and *tac4* (*tac4a* and *tac4b*) exist and up to 12 different TAC peptides have been identified up to now. Up to six TACR have been yet characterized: two TACR1 (TACR1a and TACR1b), one TACR2, and three TACR3 (TACR3a1, TACR3a2, and TACR3b). One of the 3R-duplicated *tacr2* paralogs (*tacr2b*) was lost in the teleost lineage but conserved in the eels (elopomorphs) (Campo et al. in preparation). Binding studies with the complete available tachykinin system have only been performed in the grass carp, the unique teleost species for now in which the *tac4* gene has been identified and published. In this species, *tac4b* and *tacr2b* have not yet been identified and appear in transparency in the figure. For more information, please refer to part 1 of this review. EKA, endokinin A; EKB, endokinin B; EKC, endokinin C; EKD, endokinin D; HK1, hemokinin 1; HK2, hemokinin 2; NKA, neurokinin A; NKB, neurokinin B; NKBRP, neurokinin B-related peptide; NPK, neuropeptide K; NPγ, neuropeptide gamma; SP, substance P; TAC, tachykinin peptide; *tac*, tachykinin gene; TACR, tachykinin receptor (protein); *tacr*, tachykinin receptor gene.

Vertebrate *tac* genes consist of five to seven exons that encode a pre-pro-tachykinin (PPT) peptide, named PPT-A or PPT-I for *tac1*, PPT-B or PPT-II for *tac3*, and PPT-C or PPT-III for *tac4* [for reviews (4, 32–34)]. One or two peptides are cleaved from each of the three PPT [for reviews (1)]. The TAC peptide placed close to the N terminal of PPT is called TAC-related peptide or TACRP, while the other tachykinin peptide closer to the C terminal of PPT is named TAC. For the *tac1* gene, TACRP is substance P (SP) and TAC are neurokinin A (NKA), neuropeptide K (NPK), and neuropeptide γ (NPγ), these last two being NH2-terminally extended forms of NKA. For the *tac3* gene, TACRP (only found in teleosts) is TAC3RP or neurokinin B-related peptide (NKBRP) and TAC is NKB. For the *tac4* gene, TACRP are hemokinin-1 (HK1), endokinin A (EKA), and endokinin B (EKB), while TAC are endokinin C (EKC) and endokinin D (EKD), depending on the splicing variant (**Figure 1**). Indeed in mammals, differential alternative mRNA splicing and precursor processing are observed for each of the three tachykinin genes, and as the different transcripts are regulated in a tissue-specific manner [*tac1* (35–37); *tac3* (38); *tac4* (39); for reviews (40, 41)], these mechanisms are likely to play an important role in the pleiotropic actions of the various tachykinins.

TAC are characterized by a FxGLMamide carboxy-terminus, where x is a variable, aromatic or aliphatic, amino acid [for review (5)]. Some exceptions are found: for example, the human EKC and EKD present a substitution of the final M by L (42, 43). For NKA and NKB, as well as the extended forms of NKA (NPK and NPγ), x is always a valine, leading to a FVGLM C-terminal motif (44).

#### 2.1.1 Tachykinin 1

##### 2.1.1.1 Mammals

Four peptides can be translated from the gene *tac1* (**Figure 1**): substance P (SP) or TAC1RP from exon 3; neurokinin A (NKA) or TAC1 from exon 6; neuropeptide K (NPK) or TAC1-NPK from exons 4, 5, and 6; and neuropeptide gamma (NPγ) or TAC1-NPγ from exons 3, 5, and 6 [for reviews (5, 34)]. The *tac1* gene produces four different splicing variants (α-, β-, γ-, and δ-*tac1*): *α*- and *δ*-*tac1* generate only SP; *β*-*tac1* encodes SP, NKA, and NPK; *γ*-*tac1* generates SP, NKA, and NPγ [for reviews (5, 34, 45)]. Substance P/TAC1RP was the first neuropeptide ever to be extracted in 1931 [(46); for review (47)]. In their pioneer study, von Euler and Gaddum found in extracts from horse brain and intestine an atropine-resistant factor, which induced contraction of the isolated rabbit jejunum and transient hypotension in anesthetized rabbits (46). They named this new factor substance P, with P for powder. It was only in 1971 that SP was purified and sequenced from bovine hypothalamus (48) and synthetized (49). Neurokinin A/TAC1 was discovered later in extracts of porcine spinal cord by different research groups and named differently at that time: neurokinin α (50), substance K (51), or neuromedin L (52). As SP, it was involved in ileum contraction of guinea pig (50, 52). Further analyses of the pre-pro-peptide structure revealed that peptides other than SP and NKA were encoded by the precursor and that tissue-specific alternative splicing occurred (35, 51): neuropeptide K (NPK) with 36 amino acids and neuropeptide gamma (NPγ) with 21 amino acids. Both sequences share the last 10 amino acids in the C terminal with a NKA/TAC1 sequence. NPK, isolated from porcine brain, stimulates guinea pig gallbladder contraction, plasma extravasation, hypotension, and bronchial smooth muscle spasm (53). NPγ was isolated from rabbit intestine and found to derive from γ-pre-pro-tachykinin, hence its name (54).

##### 2.1.1.2 Teleosts

The first TAC peptide to be characterized in teleosts was substance P. In 1956, a factor purified from cod brain and intestine extracts was found to have the same properties as those of mammalian substance P (55). Later, a tachykinin of 21 amino acid residues, which possesses mammalian NPγ characteristics and was named carassin, was isolated from the brain of the goldfish *Carassius auratus* (56). Then, SP and NKA were measured by radioimmunoassay in the brain of rainbow trout (57). In goldfish, Lin and Peter described two cDNAs encoding γ-PPT that may represent different transcripts resulting from the alternative transcriptional start sites and that contains the sequences of SP, carassin, and NKA (58). The *Tac1* gene was first characterized in zebrafish and found in the genomes of goldfish, medaka, and stickleback; it encodes SP and NKA (59, 60). One *tac1* gene was then found in many other teleost species, including grass carp (61). It was only recently that a second *tac1* gene, likely the result of the 3R, was identified in the grass carp; the duplicated genes were named *tac1a* and *tac1b* and shown to encode SPa and NKAa, and SPb and NKAb, respectively (62) (**Figure 1**). Before that study, it was thought that one of the duplicated *tac1* paralogs obtained by 3R was lost in teleosts [for review (1)]. Our recent bioinformatic studies revealed a wide conservation of the 3R-duplicated *tac1* genes, even those obtained by the further whole-genome duplication of the salmonids (4R) (Campo et al. in preparation).

#### 2.1.2 Tachykinin 3

##### 2.1.2.1 Mammals

The *Tac3* gene has been named *tac2* in rodents, while it is in fact an ortholog of human *tac3* (5, 63–66); so, for easier reading, rodent *tac2* will be replaced by *tac3* throughout this review. One peptide is encoded in the *tac3* gene: neurokinin B (NKB) or TAC3 (5, 32, 38, 41). NKB was purified from the extract of porcine spinal cord simultaneously by two research groups and given different names at that time: neurokinin β (50) or neuromedin K (67). As SP and NKA, it induces contraction of the guinea pig ileum (50, 67). The structure and gene organization of the neuromedin K/NKB precursor (or pre-pro-tachykinin B) was first determined in bovine (68), then in rat (69). In human, a single gene transcript encoding a single precursor and a single TAC was first revealed (70), but then three TAC3 precursors (α, β, and γ) were shown to exist (**Figure 1**) (38).

##### 2.1.2.2 Teleosts

The *tac3* gene has been characterized in a number of teleost species: zebrafish *Danio rerio* (26, 60, 71), Nile tilapia *Oreochromis niloticus* (72), goldfish *Carassius auratus* (73), striped bass *Morone saxatilis* (74), grass carp *Ctenopharyngodon idella* (62, 75), European eel *Anguilla* (76), orange-spotted grouper *Epinephelus coioides* (77), spotted sea bass *Lateolabrax maculatus* (78), and half-smooth tongue sole *Cynoglossus semilaevis* (79). While the *tac3* gene codes for only one TAC3 peptide in mammals, its ortholog in teleosts codes for two putative tachykinin peptides, TAC3 and a TAC3-related peptide [TAC3RP or NKBRP (76, 77)], earlier named neurokinin F (NKF) (71) with “F” for “fish” as it was thought to be present only in fish species and preserved along the whole teleost radiation (71, 72). As the whole-genome duplication event specific to the teleost lineage (3R) led to the duplication of the *tac3* gene into *tac3a* and *tac3b*, up to four neurokinin B peptides may exist in teleosts, namely, NKBRPa, NKBa, NKBRPb, and NKBb (**Figure 1**). Loss of *tac3b* is observed in orange-spotted grouper (77), tongue sole (79), striped bass *Morone saxatilis* (74), olive flounder *Paralichthys olivaceus*, tiger puffer *Tetraodon nigroviridis*, medaka *Oryzias latipes*, Atlantic herring *Clupea harengus*, alosa, rainbow smelt *Osmerus mordax*, and sheepshead minnow *Cyprinodon variegatus* (76), leading to the presence of only two peptides in these species (Campo et al. in preparation).

#### 2.1.3 Tachykinin 4

##### 2.1.3.1 Mammals

The molecular cloning of a mouse third PPT gene, PPT-C (later renamed *tac4*), was reported in 2000 (21). PPT-C mRNA was primarily detected in hematopoietic cells, and its derived peptide was shown to be a crucial factor for the survival of B-cell precursors and thus named hemokinin 1 [HK1 (21);]. Rat HK1 is identical to mouse HK1 (mHK1) (80). In human, the *tac4* transcript predicts two tachykinin-like peptides: one, at the N terminus, a homolog of mouse and rat HK1, was named endokinin A (HK1/EKA), and the second at the C terminus was named endokinin C (EKC), in line with their proposed peripheral endocrine roles in contrast to the neuroendocrine/neuronal role of neurokinins (39). Apart from this *tac4* transcript that was named α-*tac4*, three other splicing variants exist in human (β-, γ-, and δ-*tac4*) (**Figure 1**): β-*tac4* codes for EKB and EKD, while γ-*tac4* and δ-*tac4* encode only EKB [(39); for review (66)]. TAC4RP-EKA and TAC4RP-EKB are N-terminal extended versions of TAC4RP-HK1, with different lengths; EKB is a truncated form of EKA and EKD an N-terminally modified version of EKC (39). Thus, the *tac4* gene encodes up to five peptides in mammals: HK1, EKA, EKB, EKC, and EKD. Three of them can be translated from the TAC4-RP site and two from the TAC4 site: HK1 or TAC4RP-HK1 from exon 2; EKA or TAC4RP-EKA from exons 1 and 2; EKB or TAC4RP-EKB from exons 1 and 2; EKC or TAC4-EKC from exons 3 and 4; and EKD or TAC4-EKD from exon 4. EKC and EKD are designated tachykinin gene-related peptides [for reviews (5, 34, 40, 66)]. Interestingly, TAC4-EKC and TAC4-EKD that correspond to the TAC4 peptide have substituted the C-terminal methionine by a lysine, thus reducing or suppressing the affinity for all TAC receptors, and they differ by the length of the N terminus (39).

##### 2.1.3.2 Teleosts

It was not clear whether homologs of HK and EK were present in non-mammalian vertebrates until the *tac4* gene was identified in the genomes of various teleosts (71) and recently characterized in brain grass carp (62). The grass carp *tac4* gene encodes two mature peptides, hemokinin 1 (HK1) and hemokinin 2 (HK2) (**Figure 1**), as the mammalian *tac4*. However, the mammalian *tac4* can produce up to four different peptides depending on alternative splicing events that have not been observed in teleosts until now. HK-1 displays very weak activation for neurokinin receptors compared with HK2, likely due to a phenylalanine-to-valine substitution in the C-terminal FXGLM signature motif, leading to an inefficiency on pituitary hormone expression in grass carp pituitary cells (62). Shi and colleagues proposed that the fact that only one TAC4 isoform was isolated up to now in teleosts “might be the result of the non-functionalization by forming pseudogenes or deletion/mutations leading to the loss of redundant genes” (62). Our recent study demonstrates that the *tac4* gene has been duplicated during the 3R and two copies of the gene were conserved in most studied species (Campo et al. in preparation) (**Figure 1**).

### 2.2 Tachykinin receptors

In vertebrates, TAC peptides bind to three receptors (TACR), belonging to the first-class rhodopsin-like G-protein-coupled receptors (GPCR) (also named as family A GPCRs): TACR1, TACR2, and TACR3. These receptors are normally encoded by five exons that include the seven transmembrane domains, an extracellular N terminus enrolled in peptide recognition, and an intracellular C-terminal end in charge of the cellular response after activation of the receptor [for reviews (34, 44)].

The evolutionary scenario of tachykinin receptors in chordates (26) suggests that an ancestral tac receptor (*tacr*) gene in protochordates generated four paralogs after 1R/2R in early vertebrates (28, 29). One *tacr*, the *tacr4* gene, would have been lost before the split of the actinopterygians (ray-finned fish) and the sarcopterygians (lobe-finned fish). Further duplication of the *tacr* genes occurred during the teleost 3R. The 3R-duplicated *tacr1* and *tacr3* genes are conserved in most teleosts (Campo et al. in preparation), while one of the 3R-duplicated *tacr2* paralogs (*tacr2b*) was subsequently lost in the teleost lineage but conserved in the eels (elopomorphs) (Campo et al. in preparation). A local duplication of *tacr3a* might have occurred to give rise to *tacr3a1* and *tacr3a2* genes in the teleost lineage (26) Our recent gene search and phylogenetic study confirms this local duplication, but only in Clupeocephala, not in elopomorphs (*Anguilla* species) or osteoglossomorphs (bony tongue) (Campo et al. in preparation).

#### 2.2.1 Tachykinin receptors in mammals

Nakanishi’s group first demonstrated, by electrophysiological measurements of *Xenopus* oocytes injected with brain and stomach mRNAs, the expression of the receptors for mammalian SP (named NK1 receptor, here TACR1) and NKA (named NK2 receptor, here TACR2), respectively (81). The same year, the receptor for bovine NKA (TACR2) was cloned from bovine stomach (82). The receptors for substance P, TACR1 (83), and those for NKB, TACR3 (84), were then cloned from rat brain. When expressed in *Xenopus* oocytes and in COS cells, they can produce an electrophysiological response as follows: SP>NKA>NKB for TACR1; NKA>NKB>SP for TACR2, and NKB>NKA>SP for TACR3 (**Figure 1**) [for review (34)]. Thus, the three tachykinin receptors can bind all TAC peptides (except EKC and EKD) but with differential selectivity (44, 85–87). HK1 and EKs (EKA and EKB) exhibit the highest affinity to TACR1 (**Figure 1**) [for reviews (8, 34)].

#### 2.2.2 Tachykinin receptors in teleosts

Up to six tachykinin receptors have been characterized in teleosts (**Figure 1**), results of both whole-genome duplication and local gene duplication. In zebrafish, two 3R-duplicated *tacr1* (*tacr1a* and *tacr1b*), one *tacr2*, and three *tacr3* (*tacr3a1*, *tacr3a2*, and *tacr3b*) are identified, with *tacr3a2* arising from a local duplication of *tacr3a1* (26). In the grass carp, the same receptors are found: duplicated *tacr1* (NK1Ra and NK1Rb in the article), single *tacr2* (NK2R in the article), and three *tacr3* (NK3Ra1, NK3Ra2, and NK3Rb in the article) (62).

Using COS-7 cells that expressed zebrafish TACR3a1 (Tac3ra in the article) or zebrafish TACR3a2 (Tac3rb in the article), Biran and collaborators reported that both zebrafish NKBa and NKBRP (NKF in the article) were endogenous ligands of TACR3, while zebrafish NKBb was less effective (71). The same year, a local duplication of *tacr3a* was reported in zebrafish and binding studies of the three zebrafish TACR3 (TACR3a1, TACR3a2, and TACR3b) were investigated. NKBRPa (NKBa-13 in the article) and NKBRPb (NKBb-13 in the article) have higher potencies for inducing promoter activity of TACR3a1 and TACR3a2 in both CRE and SRE transactivation assays than NKBa-10 (26). For TACR3b, the same three NKB peptides have an inducing effect only using the SRE promoter (26). Zebrafish NKBb (NKBb-11 in the article) cannot activate any of the three TACR3s (26). In the same system, tilapia NKBRP (NKF in the article) was more effective than tilapia NKB in inducing the activity of tilapia TACR3a (Tac3ra in the article) and tilapia TACR3b (Tac3rb in the article) (72). In transfected 293-T cells, goldfish NKBa (NKBa-10 in the article), NKBRPa (NKBa-13 in the article), NKBb (NKBb-11 in the article), and NKBRPb (NKBb-13 in the article) can activate TACR3a1 (Tac3ra in the article), while TACR3b (Tac3rb in the article) can be slightly activated only by NKBa-10 (88).

In the grass carp, three studies investigated the receptor selectivity of TAC peptides, using HEK293T cells transfected with each of the six TACRs identified in this species (61, 62, 89) (**Figure 1**). The first two articles were reported before the cloning of the *tac4* gene and thus did not include TAC4 peptides (61, 89). The authors found that for TACR1 activation, the potencies were grass carp SP>NKA>NKBa>NKBRPa>NKBRPb>NKBb, and for TACR2 activation, grass carp SP, NKA, NKBa, NKBRPa, and NKBRPb had similar potency, except for NKBb which showed a low potency (61). This reveals that carp TACR2 is a multiligand receptor, which could be activated by various TACs with comparable efficacy and potency. Concerning TACR3 activation, for both TACR3a2 (NK3Ra in the article) and TACR3b (NK3Rb in the article), grass carp NKBRPb, NKBa, and NKBRPa were found to be the most effective compared with NKA, SP, and NKBb (89). Interestingly, in the third publication, HK2, product of the *tac4* gene, was shown to be able to activate all six TACRs, but with the highest activity for TACR2 (TACR2>TACR3b>TACR1a≈TACR3a1≈TACR1B>TACR3a2), while HK1 displays a very weak activation for each of TACR isoforms (62). These results suggest that while mammalian hemokinin HK1 exhibits the highest affinity for TACR1, teleost HK2 preferentially stimulates the multiligand receptor TACR2 and may thus have a similar function as other tachykinins through its activation (62).

## 3 The physiological role of tachykinins in the regulation of reproduction

**Figure 2:**
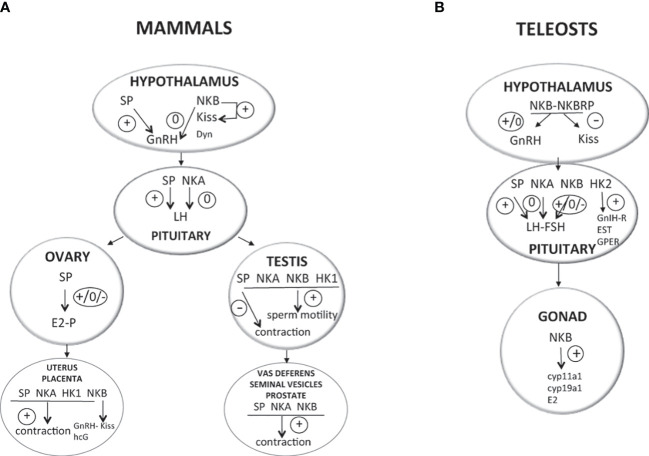
Direct effects of TAC peptides in the control of reproductive function in mammals and teleosts. TAC peptides can act directly at all the levels of the HPG axis (hypothalamus, pituitary, gonads in both mammals **(A)** and teleosts **(B)**, and other peripheral reproductive organs in mammals). At the brain level (hypothalamus), the effect of TAC on GnRH is likely kisspeptin-dependent in both mammals (KNDy neurons) and teleosts. At the pituitary level, while TAC action is only stimulatory on gonadotropins in mammals, a species specificity is observed in teleosts with either no, stimulatory, or inhibitory effects. At the peripheral level, TAC can act on ovarian steroidogenesis in mammals (either no, positive, or negative effects) and teleosts (positive effects). In mammals, a positive effect is also noted on the contraction of secondary sex organs (uterus in females; vas deferens, seminal vesicles, and prostate gland in male), as well as on sperm motility in males. For more details and for data from *in vivo* experiments, please refer to part 2 of this review and to **Table 1**. +, direct stimulatory effect; -, direct inhibitory effect; 0, no direct effect; cyp11a1, gene encoding cholesterol side-chain cleavage enzyme P450scc; cyp19a1, gene encoding aromatase; Dyn, dynorphin; E2, estradiol; ESR, nuclear estrogen receptor; FSH, follicle-stimulating hormone; GnRH, gonadotropin-releasing hormone; GnIH-R, gonadotropin-inhibitory hormone receptor; GPER, G-protein-coupled (membrane) estrogen receptor; hCG, human chorionic gonadotropin; HK1, hemokinin 1; HK2, hemokinin 2; kiss, kisspeptin; LH, luteinizing hormone; NKA, neurokinin A; NKB, neurokinin B; NPK, neuropeptide K; NPγ, neuropeptide gamma; P, progesterone; SP, substance P; T, testosterone.

Activation of the gonadotropic axis at puberty onset and maintenance of the reproductive state (with generation of GnRH pulse in mammals) are under a complex regulatory network. A major breakthrough occurred in 2003 for reproductive neuroendocrinology with the description of hypogonadotropic hypogonadism in human and mice bearing mutation in either kisspeptin gene (*kiss*) or its receptor (*kissr*) (90–92) and the following multiple studies stating that kisspeptins were the most potent secretagogues of GnRH in all mammals [for reviews (93–95)]. In addition, the involved kisspeptin neurons of the arcuate nucleus (ARC) in the hypothalamus were shown to co-express neurokinin B and dynorphin and were thus referred to as KNDy neurons, first in sheep (96) then in a variety of mammals [for review (97)]. A model was proposed in mammals for the GnRH pulse generator with NKB stimulating kisspeptin release and dynorphin inhibiting it [for reviews (98–101)]. All these data rekindle attention to other tachykinins, namely, SP and NKA, in reproductive physiology. In contrast, little is known concerning a potential involvement of tachykinin peptides derived from the *tac4* gene, hemokinin, and endokinins, in the control of reproduction, but potential actions at peripheral level are observed (**Figure 2**).

Some redundancies among the three TACR signaling pathways in the control of reproduction may occur (at least in rodents), as blockade of all three receptors (by the use of an antagonist for all three receptors) is required to inhibit LH secretion [ovariectomized (OVX) rat (104)] and the *in vitro* stimulatory effect of NKB on KNDy neurons is blocked only in the presence of a cocktail of all three receptor antagonists [male mouse (105)]. In both studies, the use of specific receptor antagonists individually has no effect (104, 105).

### 3.1 At the central level of the HPG axis

The main central targets of tachykinins on the HPG axis are the brain gonadotropin-releasing hormone (GnRH) and pituitary gonadotropins (LH and FSH). A recent review deals with the latest advances in our understanding of the biology of tachykinins in the control of GnRH release in mammals (106). However, as the HPG axis is also controlled by dopamine (DA) in teleosts, amphibians, and seasonal mammals, future studies should aim at investigating the potential regulation of dopaminergic neurons by the products of tachykinin genes. Indeed, in mammals, reports have shown interactions between tachykinins and DA at the hypothalamic level [for review (107)]. For example, Billings and colleagues demonstrated the presence of TACR3 in DA neurons in the ewe, abundantly during anestrous when DA mediates the suppression of GnRH and LH release and considerably less during the breeding season (108).

#### 3.1.1 TAC1 peptides

##### 3.1.1.1 Inactivation of TAC1 system and reproduction

To date, in humans, hypogonadotropic hypogonadism has never been associated with mutation of either the *tac1* gene or *tacr1/tacr2* genes. However, in mice, inactivation of these different genes leads to different degrees of reproductive impairments, suggesting the need for the whole tachykinin system to get full reproduction. Knockout of the *tac1* gene was obtained in female (109) and male (110) mice, which induced a delay in the onset of puberty in both sexes plus a subfertility in females (109). In contrast, mutant mice for *tacr1* are fertile (111). More recently, the characterization of a novel mouse line with congenital ablation of *tacr2* has allowed to show partially suppressed basal and stimulated LH secretion, with moderate reproductive impact (normal puberty onset and fertility), in these null mice (112). However, in the same species, impairment of TAC1 peptide action does not seem to impact reproduction, as when TACR1 or TACR2 antagonists are ip injected to 8-week-old or subcutaneously (sc) to 6-month-old female mice, no effect is observed on reproductive success or litter size (113).

##### 3.1.1.2 Effects of TAC1 peptides on GnRH expression, synthesis, and release

###### 3.1.1.2.1 Mammals

Before the description of KNDy neuron involvement in GnRH pulse generation and the renewed interest in SP and NKA as regulators of the HPG, few studies were available for a potential direct effect of TAC1 peptides on GnRH [for review (97)]. Using a perifusion system, Ohtsuka and collaborators (114) were the first to show that SP stimulated the *in vitro* release of GnRH by rat medio-basal hypothalamus (**Figure 2**). Consistent with this direct action of SP on GnRH in rodents, its receptor, TACR1, was shown to be expressed in a fourth of GnRH neurons in the mice (115), and binding sites for SP detected in the rat hypothalamus (116). In addition, SP neurons establish inputs to GnRH neurons in the rat septopreoptic area (117) as well as in human diencephalon (118) and eminence median (119). In contrast, in another mammal, the ewe, no co-expression of SP or TACR1 was detected in GnRH neurons, implying no possible direct effect of SP on GnRH in this species (120). None of GnRH neurons express TACR2 in mice (115) and no detection of NKA binding sites was observed in the hypothalamus of rats (116), suggesting a lack of potential direct effect of NKA on GnRH in both species. However, Sahu and Kalra reported in female rat that the TACR2 agonist, but not the TACR1 one, could suppress GnRH release by fragments of median eminence and arcuate nucleus in culture (121).

Apart from a potential direct action of SP and NKA on GnRH neurons *via* their respective receptors, data in mammals report effects on kisspeptin neurons, as for NKB in the KNDy network. A stimulatory action of TAC1 peptides on kisspeptin has been demonstrated in rodents. In mice, SP and NKA modulate Kiss1 neurons and kisspeptin release [SP and NKA, males (105); SP and NKA, males and ovariectomized and supplemented with E2 (OVX+E2) females (115); SP, females (109); NKA, females (122)]. SP and NKA are also able to depolarize Kiss1 neurons in male mice (105). In addition, the induction of GnRH release by icv infusion of an SP agonist in adult male and OVX+E2 mice is not observed in Kiss1r–/– mice (115). These results on the kisspeptin-dependent effect of SP on GnRH are consistent with the fact that half of kiss1 neurons express *tacr1* in this species (115). In rat, icv injection of SP elevates both *gnrh* and *kiss1* mRNA levels (123).

In other mammalian species, anatomical data suggest a possible action of TAC1 peptides on kisspeptin or GnRH, but *in vivo* studies are often lacking or report only limited action. In postmenopausal women, SP immunoreactivity is detected within Kiss1 neurons in infundibular nucleus (124) and Kiss-SP occasionally contact with GnRH in the postinfundibular eminence (119). However, to our knowledge, no *in vivo* data for SP or NKA action on GnRH *via* kisspeptin are yet available in humans. In the goat, SP fibers are also observed in close apposition within ARC KNDy (125), and high doses of TACR1 and TACR2 agonists are needed, and not effective in all individuals, to induce GnRH pulse generator activity in OVX goats (103). In the male rhesus monkey, an absence of expression of SP in kisspeptin neurons has been reported but SP fibers are observed in close apposition on ARC kisspeptin perikarya (126). In the ewe, only a small proportion of ARC kiss neurons contain *tacr1* or SP expression (120).

Recently, results in rat showed that icv injection of SP antagonizes the inhibitory effect of the mammalian ortholog of gonadotropin-inhibitory hormone (GnIH), RFRP-3, on the expression of hypothalamic *gnrh* and *kiss1* (123). These first data suggest that SP may act at different levels of the central control of HPG (GnRH, kisspeptin, or GnIH). More studies in mammals and in other vertebrates should be performed to see whether this effect of SP on GnIH action is conserved throughout evolution.

###### 3.1.1.2.2 Teleosts

In zebrafish, direct actions of TAC1 peptides are more likely on GnRH than on Kiss neurons, as associations between TAC1-immunoreactive processes and neurons for GnRH3 (the hypophysiotropic GnRH form in this species) in the ventral telencephalic area are observed, while there is no apparent proximity of TAC1 processes to kiss2 mRNA-expressing neurons in the hypothalamus (60). Recently, expression of *tacr1a* mRNA was reported in several brain regions containing GnRH3, as well as Kiss2, cells such as olfactory bulb, preoptic area, and hypothalamus (127), leading the authors to suggest that TAC1 peptides may act on both neurons. To the best of our knowledge, no functional study has been yet performed to investigate the action of TAC1 peptides on GnRH production in teleosts.

##### 3.1.1.3 Effects of TAC1 peptides on LH and FSH expression, synthesis, and release

###### 3.1.1.3.1 Mammals

A direct action of SP and NKA at the pituitary level is possible as expression of their receptors has been detected in pituitary cells, and sometimes specifically on gonadotrophs, in mammals [tacr1 (128–130); tacr2 (131)]. Moreover, SP and NKA fibers have been reported surrounding hypophyseal blood capillary vessels in the median eminence and these peptides to be present in the pituitary of mammals [NKA, rat (132); SP, rat (133–136); SP, NKA, rat (137); SP, rhesus monkey (126, 138)]. The first reported study on the *in vitro* effect of tachykinin on gonadotropins dates back to 1974. Fisher and colleagues observed that SP induced release of LH and FSH by pituitaries, from intact rats, cultured *in vitro* (139). These preliminary data using few pituitaries and a high dose of SP were followed by a contradictory one, which reported no effect of SP on LH and FSH release by hemi-pituitaries of OVX rats (140). These first data already point out the potential importance of sex steroid on SP action in the control of gonadotropins. A stimulatory effect of SP on LH release from anterior pituitary cells in culture is reported during the peripubertal period in male and female rats but not at the prepubertal age and long after maturation (141). *In vitro* perifusion of anterior pituitaries from female rats allowed to show an inhibition of GnRH-induced LH release by SP, an effect abolished by the use of the TACR1 antagonist (142). All these results obtained in rat highlight the sex steroid dependence of the *in vitro* effect of SP on gonadotropins in this species. Data are also available in another mammal, the pig. In cultured porcine gonadotrophs, SP was reported to stimulate LH release without affecting intracellular LH content (143). It also potentiated GnRH-stimulated LH release and reversed the GnRH-induced decrease of gonadotroph LH stores, these effects not being blocked by the use of the GnRH-receptor antagonist. A few years later, the same group demonstrated that the SP direct effect on pig gonadotrophs and LH release was extracellular Ca^2+^-dependent and did not involve an effect on *lhβ* transcript levels (144). Altogether, these *in vitro* data in mammals converge toward a predominantly stimulatory effect of SP on LH release (**Figure 2**). Concerning NKA, few data are available. Incubation of hemi-pituitaries with NPK and NPγ stimulates LH release in intact male rats but is not significant in castrated animals, while no significant effect is seen with NKA in both situations (145).

*In vivo* data concerning SP and NKA action on gonadotropins in mammals have been reviewed by Fergani and Navarro (97), and the following text will give a summary of them, complemented with one more recent publication (122). These *in vivo* studies, which cannot discriminate between direct and indirect effects, report diverse effects of TAC1 peptides on LH and FSH release, mostly depending on the presence or absence of sex steroids and also likely due to species differences [for review (97)]. Most of these studies reported a stimulatory effect of SP on LH release [OVX+E2 female rat (146); prepubertal female and male rats (147); normal men (148); intact female rabbit (149); OVX+E2 female mouse and intact male mouse (115); prepubertal female mouse (109); intact ewe (120)]. However, an absence of effect was sometimes observed [intact adult male rat (145); intact adult female rat (147); OVX female rat (145, 146); castrated male monkey (126); OVX or OVX+E2 female goat (103)]. Even inhibition was reported in castrated male rats (145, 150). Intravenous (iv) injections of SP failed to induce LH release in the rhesus monkey (126). However, in the cynomolgus monkey, there is a reduction in the duration and amplitude of LH surge after intragastric administrations of the TACR1 antagonist (151). In the ewe, much higher doses of SP compared with NKB are needed to stimulate LH release (120). Similarly, for NKA, various effects were obtained depending on the “sex steroid status” of the animals. Stimulation of LH release was observed in intact mature animals [male rat (145); OVX+E2 female mouse and intact male mouse (115, 147)] and in prepubertal intact male and female rats (147), while inhibition was reported in castrated animals [male rat (145); female rat (145); female mouse (115)]. An absence of effect was also sometimes seen by some authors in OVX adult female rats (121). Recently, the stimulatory action of NKA in the presence of sex steroids during adulthood in female mice was reported to be NKB-independent, as NKA was able to induce LH release in NKB-deficient mice (*tac3*KO mice) or after blockade of TACR3 by a specific antagonist (122). In addition, the stimulatory effect of NKA was kisspeptin-dependent, as it was absent in *Kiss1*KO mice (122). Interestingly, the inhibitory action of NKA on LH release in the absence of sex steroids during adulthood in female mice was found to be NKB- and dynorphin-dependent (122). In the ewe, much higher doses of NKA are needed to stimulate LH release, compared with NKB (120). In the female goat, the NKA agonist was inefficient to induce LH release either in OVX or OVX+E2 animals (103). NPK was also shown to modulate gonadotropin as icv injection of the peptide produced a suppression of LH release in ovariectomized rats (145).

A few studies addressed the effects of TAC1 peptides on FSH release in rodents. A sex difference was obtained in prepubertal rats: acute administration of the TACR1 agonist stimulated it only in females, while it was the TACR2 agonist that was able to induce it in males (147). In intact adult rats, the TACR1 agonist had no effect on FSH release in both sexes and the TACR2 one could elevate it in females (147). SP was ineffective in stimulating FSH release in OVX+E2 rats (146). In the intact male mice, central injection of TACR1 or TACR2 agonists induced an elevation of FSH secretion (115).

###### 3.1.1.3.2 Teleosts

A direct action of tachykinins at the pituitary level is also possible in teleosts as SP fibers directly innervate the pituitary (58, 152, 153). Moreover, TACR1 expression has been detected in the pituitary [zebrafish (71):; grass carp (62, 75)] and specially in LH cells [grass carp (61)]. In contrast, in grass carp pituitary, spatial distribution of TACR2 is only overlapping with prolactin cells (61, 75), and not LH cells (61). FSH cells were not investigated in these studies (61, 75).

To date, only one study investigated the direct effects of peptides encoded by the *tac1* gene on teleost pituitary hormone expression and release [**Figure 2** (61)]. Using primary culture of prepubertal grass carp pituitary cells, Hu and collaborators showed that grass carp SP and NKA could elevate prolactin (*prl*) and somatolactin-α (*slα*) mRNAs and hormone secretion, without any effect on proopiomelanocortin (*pomc*), *fshβ*, thyrotropin *β* (*tshβ*), glycoprotein α-subunit (*gp-α*), growth hormone (*gh*), and *slβ* expression. For LH, SP but not NKA could induce a dose-dependent inhibition of *lhβ* mRNA levels (after 24 h of treatment), while both peptides induced a dose-dependent stimulation of LH release with a lower potency and efficacy for NKA (after 3 h). This induction of LH release by SP and NKA and the inhibition of *lhβ* mRNA by SP were blocked by the use of the TACR1 antagonist but not of TACR2 or TACR3 antagonists, in agreement with the fact that TACR1 was the only form of TACRs detected in grass carp gonadotrophs. Moreover, SP was able to partially suppress GnRH induction of *lhβ* mRNA, while co-treatment with the TACR1 antagonist enhanced this induction. TAC1 peptides in grass carp can thus have differential effects on LH release and *lhβ* mRNA levels *via* activation of TACR1 in gonadotrophs. More studies in other teleost species are needed to decipher whether these actions are species-specific or common to all teleosts.

#### 3.1.2 TAC3 peptides

##### 3.1.2.1 Inactivation of the TAC3 system and reproduction

In 2003, Pintado and collaborators injected intraperitoneally an antagonist of TACR3, the preferential receptor for TAC3 peptides, to 8-week-old female rats and showed no effect on reproductive success or litter size, while a 6-month-old subcutaneous injection of the same antagonist resulted in a reduction in the litter size (113). Later, mutations in the *tac3* or *tacr3* genes were characterized which lead to hypogonadotropic hypogonadism in human [(154–159); for review (160)], which could be reversed in adulthood (157). Similarly, in mice, *tac3* (161) or *tacr3* (162) null females show delayed sexual maturation and abnormal estrous cyclicity, which recover in adulthood leading to fertility, although they produced fewer pups per liter. In contrast, timing of sexual maturation and fertility are preserved in *tac3* (161) or *tacr3* (162) null males. In a teleost, the zebrafish, the knockout of either *tac3a*, *tac3b*, or both does not disrupt the reproduction (spermatogenesis and folliculogenesis are not impaired) (163). The impact of *tac3* gene mutation should be now studied in other teleost species, as knockout studies of reproductive genes such as the different types of *gnrh* [*gnrh3* (164); *gnrh2* (165)] and *kiss* [*kiss1*, *kiss2*, *kissr1*, and *kissr2* (166)] system genes all generate zebrafish with normal gametogenesis, suggesting that this species may have a high compensatory mechanism [for reviews (167, 168)] and may not reflect the situation observed in all teleosts.

##### 3.1.2.2 Effects of TAC3 peptides on GnRH expression, synthesis, and release

###### 3.1.2.2.1 Mammals

In mammals, the first analysis of *tacr3* expression was performed in rodents and showed its presence in GnRH neurons, indicating a possible direct effect of TAC3 on GnRH [rat (169); mouse (170)]. However, an absence of direct regulation of GnRH release by NKB was demonstrated using hypothalamic explants from adult male mice (171). In addition, in the same *in vitro* system, NKB was able to completely abolish the stimulation of GnRH release induced by kisspeptin (171). These results in mice suggested that NKB could regulate GnRH only *via* an action on kisspeptin (**Figure 2**), which is in agreement with the more recent demonstration of a minimal expression of *tacr3* in GnRH neurons, but its expression on virtually all KNDy neurons, in this species (115, 172). In female sheep, no TACR3 immunoreactivity was revealed in GnRH neurons, but GnRH neurons and fibers were in proximity to NK3R-containing ones (173). Use of the immortalized GT1-7 cell line, which represents mature post-migratory GnRH neurons with expression of TACR3, allowed to show differential effects on GnRH release depending on the length of exposure: acute treatment with NKB increases GnRH secretion, while long-term treatment decreases it by repressing transcription (174). In the arcuate nucleus of the hypothalamus, TAC3 is co-expressed with kisspeptin and dynorphin in the so-called KNDy neurons [for review (98)]. This was first demonstrated in sheep (96). KNDy neurons project to GnRH neurons and positively regulate their activity, being responsible for the generation of GnRH pulsatility in the hypothalamus of mammals [for reviews (97, 100, 175)]. Ablation of these neurons in female rats induces hypogonadotropic hypogonadism (176). Most of the studies show that neurons of ARC and particularly KNDy neurons project to the axonic terminals of GnRH neurons (98). Therefore, it is possible to assume that KNDy neurons might act on those terminals in a direct manner or using intermediate neurons to regulate the GnRH release (175). The most accepted hypothesis for mammalian KNDy neurons proposes that TAC3 acts in a positive manner and that Dyn acts in a negative way on the pulsatile release of kisspeptin by KNDy neurons (172, 177). In the ewe also, a high percentage of kisspeptin neurons produces dynorphin and NKB (96). A recent review addresses the question whether the KNDy model for the control of GnRH pulses applies to humans and other primates, compiling data showing that colocalization of kisspeptin and NKB is also observed in rhesus monkeys and humans (178). In addition, the ability of kisspeptin to induce LH release in patients with mutations in TAC or TACR3 tends also toward a proximal action of NKB to kisspeptin in stimulating GnRH secretion (179).

*In vivo* studies in different mammals have shown the stimulatory effect of NKB on GnRH secretion [prepubertal and pubertal rhesus monkeys: female (180) and male (181); ewe (182); goat (103, 168)]. Electrophysiological studies showed that icv administration of the TACR3 agonist (senktide) suppressed the GnRH pulse generator in OVX rats (183), while it induced GnRH release in intact male mice (184).

###### 3.1.2.2.2 Teleosts

In tilapia, Mizrahi and colleagues investigated the co-expression of the three different forms of GnRH present in this species with TACR3s. They show that GnRH3 neurons expressed *tac3ra*, but not *tac3rb*, while the contrary was observed for GnRH2 neurons, and GnRH1 ones expressed both *tacr3* (185). In the striped bass, TAC3 peptides, NKB, and NKBRP (NKF in the article) have an inconsistent effect (no or stimulatory only at the highest dose) on *gnrh1* expression by brain slices in culture, while persistently downregulating *kiss2* expression (74) (**Figure 2**).

In contrast to human and rodents, the expressions of tachykinins and kisspeptins are not always expressed in the same neurons in teleosts. In the zebrafish, NKB/TAC3 and NKF/NKBRP/TAC3RP are expressed in the *nuclear lateralis tuberis* (NLT), which is the teleost homologous structure to the ARC (71), but *kiss2* expression has not been found in this area (186). This finding in zebrafish, however, does not exclude that TAC neurons project on kisspeptin ones. In the striped bass, NKB neurons innervate the largest kiss2 neuronal population in the hypothalamus, which also expresses TACR3, while no expression of TACR3 or no NKB neuronal projection is detected for GnRH1 soma (74). In addition, in this species, TAC3 peptides, NKB and NKBRP (NKF in the article), are able to downregulate *kiss2* gene expression *in vivo*, while having no effect on *gnrh1* expression (74). In addition, cotreatment with a NK3R antagonist abolishes the negative effect of TAC3 peptides on *kiss2* mRNA levels (74). These results in the striped bass suggest that tachykinin peptides may act preferentially on the kisspeptin system, as in mammals. When injected to goldfish females in mid-vitellogenesis and males in late-spermatogenesis, three NKB peptides (NKBa-13, NKBa-11, and NKBb-13), but not the fourth one (NKBb-11), increase hypothalamic *gnrh3* mRNA levels (73). NKBa-10 and NKBa-13 ip injected to goldfish females at the early vitellogenic oocyte stage and males at the early spermatogenesis stage decrease mRNA levels of both hypothalamic *kiss2* and *gnrh3* (except NKBa-13 on *gnrh3*) (88). In tilapia, NKBRP injected to mature male tilapia inhibits the expression of brain *gnrh-I* and *kiss2*, while NKB has no effect (187). A recent study in the Japanese eel, *Anguilla japonica*, reports that ip injection of each of the four mature peptides found in this species gives different effects depending on the peptides and the doses used: a low dose of the four peptides had no effect on neither *gnrh1* (*mgnrh*) and *gnrh2* (*cgnrh*) expression, while a high dose of NKBa-10 and NKBb-13 (and not NKBa-13 and NKBb-10) stimulates *gnrh1* expression (188). All these data demonstrate that different regulations of *gnrh* and *kiss* expression by TAC3 peptides (from none to stimulatory or inhibitory effects) may be encountered among teleosts, depending on the species, the maturity stage, the doses, and the peptides tested.

The non-systematic action of TAC3 peptides on GnRH and kisspeptin in teleosts, compared with the situation observed in mammals, is likely due to the surprisingly non-essential character of these two neuropeptides for reproduction in some teleost species. Indeed, recent knockout studies demonstrated that *gnrh3* and *gnrh2* in zebrafish (164, 189), *gnrh1* in male medaka (190), and *kiss1* and *kiss2* in zebrafish (166) and medaka (191) were dispensable for normal reproductive function. In zebrafish, even triple mutants for *gnrh3*, *kiss1*, and *kiss2* undergo normal puberty and gonad maturation (192). This lack of effect on reproduction of GnRH and kisspeptin gene editing led many scientists to make assumptions on the possibility of physiological compensatory phenomena in teleosts (for reviews: 167, 193–196). In addition to these knockout results, many data are available, stating reproductive actions of GnRH (for review: 194) and kisspeptins at various HPG levels in teleosts (for reviews: 195, 196).

##### 3.1.2.3 Effects of TAC3 peptides on LH and FSH expression, synthesis, and release

###### 3.1.2.3.1 Mammals

A direct action of tachykinins at the pituitary level is possible as *tacr3* expression has been detected in this gland in mammals [ewes (197); pigs (198), and gilts (199)]. Moreover, NKB fibers have been reported surrounding hypophyseal blood capillary vessels in the median eminence [monkeys (200)]. To the best of our knowledge, only one *in vitro* study has investigated the potential direct effect of NKB on gonadotropins in mammals, by using a gonadotroph cell line (201). The authors reported no effect of NKB on *lhβ* and *fshβ* mRNA expression, even if TACR3 was detected in this cell line.

Comparing various tachykinins *in vivo*, Sahu and Kalra (121) were the first to report that NKB-containing implants, in the third ventricle of OVX rat brain, did not induce any change in LH release. Later, this absence of NKB effect on LH was also shown after either ip or icv administration to intact adult male mice (171). However, evidence for stimulatory effects of NKB on LH has since been documented in many mammalian species [for reviews (97, 106, 178)], as for example in prepubertal female rats (16, 202). In some studies, the stimulatory effect of NKB on LH is only observed under physiological sex steroid levels (i.e., intact or OVX+E2 adult animals) [adult male and female mice (115, 203); adult male and female rats (203, 204); lactating female cattle (205)]. In contrast, in monkeys, NKB is able to stimulate LH release in castrated juvenile (200, 206) and adult (207) males. In the sheep, castration does not prevent the stimulatory action of NKB in adult females (207, 208), as compared with intact females [adult (108, 209); prepubertal (210)]. A similar situation is observed in the female goat with a stimulatory effect of the iv administered NKB agonist (senktide) (103) or no effect of the icv injected NKB (177), regardless of the gonadal status. Recently, iv administration of senktide has even been shown to be efficient in stimulating LH release in fetal male and female sheep (211). In humans, early studies report no gonadotropin-stimulating effect of NKB iv administered in adult men and women (212, 213), but a series of data obtained by Skorupskaite and collaborators using the TACR3 antagonist given orally show a decrease in overall circulating LH levels and LH pulsatility in adult men (214) and women (215–217). Few studies report an inhibitory action of senktide on LH release, regardless of the steroid milieu in female rat (183, 218) or only in the absence of sex steroids in female mice (172).

Concerning FSH, either stimulatory [mouse (115, 147, 219); monkey (207); man (214, 220)] or no effect [mouse (171); rat (147); woman (212, 214, 220); man (212, 213)] of NKB has been reported.

These various effects on LH and FSH in mammals could be due to species, physiological status, or mode of peptide administration. **Table 1** gives details on all these *in vivo* studies of NKB action on gonadotropins.

**Table 1:** *In vivo* studies concerning NKB action on gonadotropins in mammals and teleosts.

Species	Sex	Age	Gonadal status	Treatment	Route	Effect	References
**Mammals**							
Human	Man	Adult		NKB	iv	None on LH-FSH release	Jayasena et al (34)
	Woman	Adult		NKB	iv	None on LH-FSH release	Jayasena et al (34)
	Man	Adult		NKB	iv	None on LH-FSH release	Narayanaswamy et al (35)
	Women	Adult		TACR3 antagonist	Orally	Inhibitory on LH release	Skorupskaite et al (36)
	Man	Adult		TACR3 antagonist	Orally	Inhibitory on LH-FSH release	Skorupskaite et al (37)
	Woman	Adult		TACR3 antagonist	Orally	Inhibitory on LH release	Skorupskaite et al (38)
	Woman	Adult	Postmenopausal	TACR3 antagonist	Orally	Inhibitory on LH release None on FSH release	Skorupskaite et al (39)
Rhesus monkey *Macaca mulatta*	Male	Juvenile	Agonadal	NKB Senktide	iv	Stimulatory on LH release	Ramaswamy et al (40)
Rhesus monkey	Male	Juvenile	Agonadal	Senktide	iv	Stimulatory on LH release	Ramaswamy et al (41)
Cynomolgus monkey *Macaca fascicularis*	Male	Adult	ORX	TACR3 antagonist	Orally	Inhibitory on LH-FSH release	Fraser et al (42)
Rat	Female	Adult	OVX	NKB	icv	None of LH release	Sahu and Kalra (43)
	Female	Adult	OVX+E2	Senktide	icv	Inhibitory LH release	Sandoval-Guzman et al (44)
	Female	Adult	Intact	Senktide	icv	Stimulatory on LH release	Navarro et al (45)
			OVX+E2	Senktide	icv	Stimulatory on LH release	Navarro et al (45)
			OVX+Sham	Senktide	icv	Inhibitory on LH release	Navarro et al (45)
	Female	Prepubertal	Intact	Senktide	icv	Stimulatory on LH release	Navarro et al (45)
	Female	Adult	Intact	Senktide	icv	Stimulatory on LH release	Navarro et al (45)
	Female	Prepubertal	Intact	TACR3 antagonist	icv	Decreasing trend on LH release	Navarro et al (45)
	Female	Adult	OVX	Senktide	icv	Inhibitory LH release	Kinsey-Jones et al (46)
		Adult	OVX+E2	Senktide	icv	Inhibitory LH release	Kinsey-Jones et al (46)
	Female	10-days	Intact	Senktide	icv	Stimulatory on LH release	Ruiz-Pino et al (47)
		25-days	Intact	Senktide	icv	Stimulatory on LH release	Ruiz-Pino et al (47)
		30-days	Intact	Senktide	icv	Stimulatory on LH release	Ruiz-Pino et al (47)
		36-days (Pubertal)	Intact	Senktide	icv	Stimulatory on LH release	Ruiz-Pino et al (47)
		Adult	Intact	Senktide	icv	Stimulatory on LH release	Ruiz-Pino et al (47)
		Adult	OVX+T	Senktide	icv	Stimulatory on LH release	Ruiz-Pino et al (47)
		Adult	OVX+Sham	Senktide	icv	None on LH release	Ruiz-Pino et al (47)
	Male	10-days	Intact	Senktide	icv	Stimulatory on LH release	Ruiz-Pino et al (47)
		25-days	Intact	Senktide	icv	Stimulatory on LH release	Ruiz-Pino et al (47)
		30-days	Intact	Senktide	icv	Stimulatory on LH release	Ruiz-Pino et al (47)
		45-days (Pubertal)	Intact	Senktide	icv	None on LH release	Ruiz-Pino et al (47)
		Adult	Intact	Senktide	icv	None on LH release	Ruiz-Pino et al (47)
		Adult	ORX+E2	Senktide	icv	None on LH release	Ruiz-Pino et al (47)
		Adult	ORX+Sham	Senktide	icv	Inhibitory on LH release	Ruiz-Pino et al (47)
	Female	Prepubertal	Intact	Senktide	icv	Stimulatory on LH release	Grachev et al (48)
	Female	Adult	OVX+E2	Senktide	icv	Inhibitory on LH release	Grachev et al (48)
		Adult	OVX+E2	Senktide +Antagonist	icv	Blockade of inhibitory on LH release	Grachev et al (48)
	Female	10-days	Intact	Senktide	icv	Stimulatory on FSH release	Ruiz-Pino et al (49)
		25-days	Intact	Senktide	icv	Stimulatory on FSH release	Ruiz-Pino et al (49)
		36-days (Pubertal)	Intact	Senktide	icv	None on FSH release	Ruiz-Pino et al (49)
		Diestrus 1	Intact	Senktide	icv	None on FSH release	Ruiz-Pino et al (49)
		Proestrus	Intact	Senktide	icv	None on FSH release	Ruiz-Pino et al (49)
		Adult	OVX	Senktide	icv	None on FSH release	Ruiz-Pino et al (49)
	Male	10-days	Intact	Senktide	icv	Stimulatory on FSH release	Ruiz-Pino et al (49)
		25-days	Intact	Senktide	icv	None on FSH release	Ruiz-Pino et al (49)
		30-days	Intact	Senktide	icv	None on FSH release	Ruiz-Pino et al (49)
		45-days (Pubertal)	Intact	Senktide	icv	None on FSH release	Ruiz-Pino et al (49)
		Adult	Intact	Senktide	icv	None on FSH release	Ruiz-Pino et al (49)
		Adult	ORX	Senktide	icv	None on FSH release	Ruiz-Pino et al (49)
Mouse	Female	Adult	OVX+E2	Senktide	icv	None on LH release	Navarro et al (50)
		Adult	OVX+Sham	Senktide	icv	Inhibitory on LH release	Navarro et al (50)
	Male	Adult	Intact	NKB	ip	None on LH release	Corander et al (51)
	Male	Adult	Intact	Senktide	icv	Stimulatory on LH-FSH release	Navarro et al (52)
	Male	Adult	Intact	Senktide	icv	Stimulatory on LH-FSH release	Navarro et al (53)
	Female	Adult	OVX+E2	Senktide	icv	Stimulatory on LH-FSH release	Navarro et al (53)
		Adult	OVX+Sham	Senktide	icv	Inhibitory on LH release	Navarro et al (53)
Sheep	Female	Adult	Anestrous	Senktide	icv	Stimulatory on LH release	Billings et al (54)
		Adult	Follicular phase	Senktide	icv	Stimulatory on LH release	Billings et al (54)
		Adult	Luteal phase	Senktide	icv	None on LH release	Billings et al (54)
	Female	Prepubertal		Senktide	iv	Stimulatory on LH release	Nestor et al (55)
	Female	Adult	Anestrous	NKB	icv	Stimulatory on LH release	Sakamoto et al (56)
	Female	Adult	OVX	TACR3 antagonist	Microimplants	Inhibitory on LH release	Goodman et al (57)
	Female	Adult	OVX	TACR3 antagonist	iv	Inhibitory on LH release = prolongs LH interpulse interval	Fraser et al 5 (42)
	Female	Adult	Luteal phase	Senktide	icv	Stimulatory on LH release	Li et al (102)
			OVX	TACR3 antagonist	icv	Inhibitory on LH release	Li et al (102)
	Female	Fetal		Senktide	iv	Stimulatory on LH release	Amodei et al (58)
	Male	Fetal		Senktide	iv	Stimulatory on LH release	Amodei et al (58)
Cattle	Female		Lactating	Senktide	iv	Stimulatory on LH release	Nakamura et al (59)
Goat	Female	Adult	OVX	NKB	icv	None on LH release	Wakabayashi et al (60)
			OVX+E2	NKB	icv	None on LH release	Wakabayashi et al (60)
			OVX	Senktide	iv	Stimulatory on LH release	(103)
			OVX+E2	Senktide	iv	Stimulatory on LH release	(103)
**Teleosts**							
Zebrafish	Female	Adult	Mature	zfNKBa zfNKBb zfNKBRP (NKF)	ip	Stimulatory on LH release Stimulatory on LH release Stimulatory on LH release	Biran et al (61)
Tilapia	Male	Adult	Mature	tiNKB tiNKBRP (NKF)	ip	Stimulatory on LH-FSH release Stimulatory on LH release	Biran et al (62)
	Female	Adult	Mature	tiNKB tiNKBRP	ip	None on *lh*-*fsh* mRNAs None on *lh*-*fsh* mRNAs	Jin et al (63)
	Female	Adult	Mature	ti NKB tiNKBRP (NKF) NKB analog NKBRP analog	ip	Stimulatory on LH release Stimulatory on *lh*-*fsh* mRNAs Stimulatory on LH release None on *lh*-*fsh* mRNAs Stimulatory on LH-FSH release Stimulatory on *lh*-*fsh* mRNAs Stimulatory on LH-FSH release Stimulatory on *lh* mRNAs None on *fsh* mRNAs	Mizrahi et al (64)
Goldfish	Female	Adult Sexually immature	Early vitellogenesis	gfNKBa-10 gfNKBa-13	ip	Inhibitory on *lh*-*fsh* mRNAs Inhibitory on *lh*-*fsh* mRNAs	Liu et al (65)
			Mid-vitellogenesis	gfNKBa-10 gfNKBa-13 gfNKBb-11 gfNKBb-13	ip	Stimulatory on *lh* mRNAs None on *fsh* mRNAs Stimulatory on *lh*-*fsh* mRNAs None on *lh*-*fsh* mRNAs Stimulatory on *lh*-*fsh* mRNAs	Qi et al (66)
	Male	Adult Sexually immature	Early spermatogenesis	gfNKBa-10 gfNKBa-13	ip	Inhibitory on *lh*-*fsh* mRNAs Inhibitory on *lh* mRNAs None on *fsh* mRNAs	Liu et al (65)
			Late spermatogenesis	gfNKBa-10 gfNKBa-13 gfNKBb-11 gfNKBb-13	ip	Stimulatory on *lh*-*fsh* mRNAs Stimulatory on *lh*-*fsh* mRNAs None on *lh*-*fsh* mRNAs Stimulatory on *lh*-*fsh* mRNAs	Qi et al (66)
Orange-spotted grouper	Female	Adult	Early vitellogenesis	grouperNKB grouperNKBRP	ip ip	Stimulatory on *lh* mRNAs None on *fsh* mRNAs None on *lh*-*fsh* mRNAs	Chen et al (67)
Japanese eel	Female	Silver stage (prepubertal stage)	Immature	eelNKBa-10 eelNKBa-13 eelNKBb-10 eelNKBb-13	ip	Stimulatory on *lh* and *fsh* mRNAs None on *lh*-*fsh* mRNAs None on *lh*-*fsh* mRNAs Stimulatory *fsh* mRNAs None on *lh* mRNAs	Zuo et al (68)

###### 3.1.2.3.2 Teleosts

TACR3s (*tac3ra1*, *tac3ra2*, and/or *tac3rb*) are expressed at the pituitary level in various teleosts [zebrafish (26, 71); spotted sea bass (78); grass carp (62, 89, 221, 222)] and in both LH and FSH cells in tilapia (72), making a direct effect of NKB and NKBRP possible on gonadotropin synthesis and release. Interestingly, in the grass carp, Xu and collaborators reported that *tacr3a* is expressed in both LH and somatolactin α (SLα) cells, while *tacr3b* expression is only found in SLα cells (89). FSH cells were not investigated in this study (89). In tilapia, when NKB and NKBRP are applied to mature male pituitary cells, they both increase FSH and LH release (72). In culture of pituitaries from mixed sexed juveniles of this species, NKBRP downregulates *fshβ* and *lhβ* mRNAs, while NKB has no effect (187). Still in tilapia, Mun and colleagues recently compared responses of pituitary cells and pituitaries to NKB and NKF in males and females. They reported that expressions of *fshβ* and *lhβ* mRNAs did not show any change after treatment of whole pituitaries with NKB or NKF in both sexes (223). In contrast, the use of primary culture of pituitary cells allowed them to find that NKB could stimulate *fshβ* and *lhβ* mRNAs in female and inhibit them in male, while the contrary was observed with NKF (223). These results highlight major differences according to the maturation stage, to the protein or mRNA, or to the type of culture (primary cell culture versus organotypic culture) concerning the effects TAC3 peptides have on gonadotropin in tilapia. The other studies available suggest in addition to species differences, using the same method, primary cultures of pituitary cells (**Figure 2**). In the striped bass, the effects of NKB and NKBRP were stimulatory on LH and FSH release but absent on their mRNAs (74). In the European eel, the four peptides encoded by the *tac3* gene were able to inhibit *lhβ* mRNAs by pituitary cells in culture but had no effect on *fshβ* mRNAs (76). Some other studies, using this cell culture system, reported no effect of NKB and NKBRP on *fshβ* and *lhβ* mRNAs [grass carp (75); orange-spotted grouper (77)].

In teleosts, most of the *in vivo* data showed an increase of gonadotropin release and expression after treatment with TAC3 peptides. In zebrafish, homologous (zebrafish) NKBa and NKBRP (NKF in the article) induce LH release when injected to mature females (71). Among the four neurokinin B peptides characterized in zebrafish, NKBb presents a modified C-terminal motif from the typical tachykinin FVGLM to FVGLL, thus losing the final methionine. This change leads to a decreased affinity of this peptide for the two TACR3 and a highly reduced *in vivo* effect, compared with other neurokinin peptides (71, 73). However, the finding of a third TACR3 in the zebrafish genome (26) increases the chances that the NKBb with a different C-terminal motif may be active. In tilapia, ip injections of homologous NKB to mature males increase both FSH and LH plasma levels, while homologous NKBRP induces only LH release (72). In mature female tilapia, ip injection of tilapia NKB and NKBRP has no effect on pituitary *lhβ* and *fshβ* mRNA levels (185). More recently, Mizrahi and colleagues developed specific NKB and NKBRP (NKF in the article) analogs based on the structure of the mammalian NKB analog, senktide (185). When ip injected to mature female tilapia, these analogs increase plasma LH levels as native (tilapia) NKB and NKBRP do, and they are even able to increase FSH release while native ones have no effect (185). Concerning mRNA levels, native NKB and NKB analogs are efficient in stimulating *lhβ* and *fshβ*, whereas native NKBRP has no effect and NKBRP (NKF in the article) analog stimulates only *lhβ* (185). When injected to goldfish females at mid-vitellogenesis and males at late spermatogenesis, homologous NKBa-10, NKBa-13, and NKBb-10, but not NKBb-11, increase pituitary *lhβ* mRNA levels (73). An increase in *fshβ* mRNA levels is observed in females only after administration of goldfish NKBa-13 and NKBb-13, while in males all peptides, except NKBb-11, induce these levels (73). In sexually immature goldfish, NKBa-10 and NKBa-13 ip injected to females at the early vitellogenic oocyte stage and males at the early spermatogenesis stage decrease mRNA levels of both pituitary *lhβ* and *fshβ* (88). In the female orange-spotted grouper *Epinephelus coioides* at early vitellogenic stages, ip injection of NKB increases pituitary *lhβ*, but not *fshβ*, mRNA levels, while administration of NKBRP has no effect on these expressions (77). A recent study in the Japanese eel reports that ip injection of each of the four mature peptides found in this species gives different effects depending on the peptides and the doses used: a high dose of the four peptides inhibits *lhβ* and *fshβ* expression, while a low dose of NKBa-10 and NKBb-13 stimulates them (188). All these data show various effects of TAC3 peptides on gonadotropin release and expression among teleosts, depending on the species, the maturity stage, the doses, and the peptides tested. **Table 1** compiles all these *in vivo* studies of NKB action on gonadotropins.

#### 3.1.3 TAC4 peptides

Little is known on the effects of TAC4 peptides, HK and EK, on the central reproductive brain–pituitary axis. To the best of our knowledge, the only available study was realized in a teleost, the grass carp. Using transcriptomic analysis of TAC4 peptide effects on pituitary cells, Shi and colleagues have recently demonstrated that HK2 downregulates the pituitary expression of one GnIH receptor (GnIHR3) and five estrogen receptors (nuclear: ESR1, ESR2a, ESR2b, and ESRRβ; membrane: GPER1), while upregulating the expression of another nuclear estrogen receptor (ESRRγ) (62) (**Figure 2**). In mammals, the known reproductive role of HK1 takes place at the peripheral level (33), while up to now, none is attributed to EKs.

### 3.2 At the peripheral level

Tachykinins can also act at the levels of the gonads and the secondary sex organs, *via* paracrine and autocrine effects, in both females and males (**Figure 2**).

#### 3.2.1 Effects on the female reproductive system

##### 3.2.1.1 Mammals

A review has already been dedicated to tachykinin involvement in mammalian ovarian function (224), and the text below is only a summary. Expression of *tac1* and *tac3* (mRNA), as well as their receptors, is detected in the mammalian ovary, oocytes, and granulosa cells (113, 224), indicating potential autocrine/paracrine effects. Isolated cumulus granulosa cells in mice express *tac1*, *tac3*, and *tac4* (113). The control of ovarian steroid secretion by tachykinins in mammals has been previously reviewed (224). Briefly, data were obtained in various mammalian species with different results. In rats, exposure of granulosa cell culture or ovarian fragment in culture with SP and SP analog was unable to modify estrogen or progesterone release (225). In hamster, depending on the age of the animals, treatment of ovaries in culture with SP could stimulate (15-day-old hamsters) or inhibit (adult hamsters) or have no effect (neonatal hamsters) on estradiol release (226). Concerning progesterone release, in the same culture system, SP has a stimulatory effect (neonatal and adult hamsters) or no (15-day-old hamsters) effects (226). Using luteal cells in culture and exposure to SP, opposite results were obtained on progesterone release in basal conditions or under stimulation with LH in two different artiodactyla/ungulata species: stimulation in bovine (227) and inhibition in pig (228). In pig, SP treatment did not change estradiol release by granulosa cells in culture but stimulated it by luteal cells (228). These various effects of SP on *in vitro* ovarian sex steroid release in mammals thus likely depend on species, type of cells, and age of animal.

Genes encoding tachykinin peptides (*tac1*, *tac3*, and *tac4*) and receptors *(tacr1*, *tacr2*, and *tacr3*) are all expressed in the uterus of mice (45, 113, 229), rats (230), and humans (231, 232), suggesting potential autocrine/paracrine effects. Their expressions change during the estrous cycle and during pregnancy [mouse (45):; rat (233, 234)]. TACR2 is involved in human uterine contraction and is regulated during pregnancy (33). An altered expression of SP, NKA, and HK1 and their receptors is observed in uterine leiomyomata in human (235). When applied to isolated myometrium from non-pregnant women, SP, NKA, and NKB produce contractions, while the TACR2 receptor-selective antagonist abolishes the uterotonic effect of the NKA agonist (231). These TAC peptides also produce a direct contractile effect on uterine smooth muscle in mice (5, 236), rats (229, 234, 237), and pregnant women (231, 238). Human HK1 is also a uterine stimulant in humans (33).

In the rat placenta, downregulation of *tac3* and *tacr3* expression is associated with pregnancy (41). NKB placental levels are increased at term labor in women (239). In the placenta of preeclampsia women, elevated circulating NKB and increased *tac3* expression are reported as compared with placenta of normal pregnant women (41, 70, 240). TAC3 and TACR3 may contribute to preeclampsia during late pregnancy (41, 241). NKB stimulates the expression of *gnrh*, *kiss*, and human chorionic gonadotropin (hCG) by primary cultures of rat placental cells (242).

##### 3.2.1.2 Teleosts

An autocrine/paracrine action is also possible in teleosts as both NKB and NK3R are expressed in the ovary [zebrafish (26, 71); tilapia (72)]. In the zebrafish, a direct effect of neurokinin B on the ovary is reported, as it stimulates estradiol production and increases the expression of cyp11a1 and cyp19a1 in primary cultures of follicular cells (243).

#### 3.2.2 Effects on the male reproductive system

##### 3.2.2.1 Mammals

The involvement of tachykinins in the regulation of mammalian testicular function has already been reviewed (236, 244, 245), and the text below is a brief summary.

SP inhibits testosterone production and release by isolated Leydig cells in hamster (246, 247). *Tac1*, *tac3*, and *tac4* genes are expressed in the human sperm (248). Tachykinins are likely to enhance the sperm motility by TACR1- and TACR2-dependent mechanisms, as TACR1- and TACR2- (but not TACR3-) selective antagonists can reduce the stimulating effect of phosphoramidon in human (248). Human HK1 also promotes progressive sperm motility (**Figure 2**) with a potency similar than that of NKA (lower than that of SP and higher than that of NKB) (249). All classical TACRs seem to be involved in these actions, but the role of TACR1 was predominant (249).

Tachykinins also stimulate contractility of the *vas deferens* and of seminal vesicles [**Figure 2**, for review (236)]. SP (TAC1RP) and NKA (TAC1) are present in the prostate of guinea pig and rat at low levels, of dog abundantly and absent in human prostate [for review (236)], while *tac1, tac3*, and *tac4* mRNA expressions have been detected in human prostate (39, 232). Prostate contraction by tachykinins in human involves TACR2 (250).

##### 3.2.2.2 Teleosts

An autocrine/paracrine action is also possible in teleosts as both NKB and NK3R are expressed in the testis [zebrafish (71); tilapia (72)]. In tilapia, recent use of NKB antagonists by ip injections on adult males reduced the number of spermatozoa, leading to lower fertility (251), an effect which can be direct as male tilapia have significant amounts of TACR3 in the testis (72).

## 4 The physiological role of tachykinins in the regulation of food intake

As described previously in this review, TAC peptides (SP and NKA) were discovered for their contractile role on the gastrointestinal tract (GIT) in mammals. However, far less direct evidence is available concerning the regulation of food intake by tachykinin peptides when compared with their role in reproduction. Nevertheless, recent data, notably in two teleosts, the sea bass *Dicentrarchus labrax*, and the grass carp, highlight a potential major regulatory role of TAC3 and TAC4 peptides in the regulation of genes involved in feeding and gut motility.

**Figure 3:**
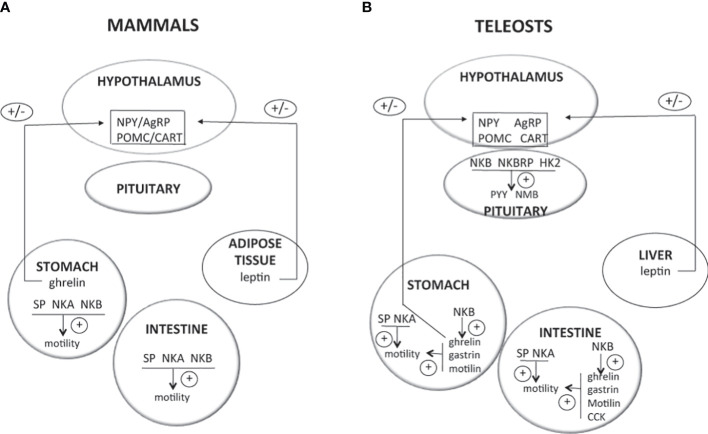
Direct effects of TAC peptides in the control of food intake and gut motility in mammals and teleosts. TAC peptides can act directly at different levels (hypothalamus, pituitary, and gastrointestinal tract) to influence food intake and gut motility. In mammals **(A)**, most of the available studies report the stimulatory effects of tachykinins on gut motility. In teleosts **(B)**, recent *in vitro* studies are emerging, showing direct effects of TAC3 and TAC4 peptides on the expression of neuropeptides highly expressed in the pituitary and that are involved in the central control of food intake. These TAC peptides can also influence the expression of genes from the gut that control its motility. For more details and for data from *in vivo* experiments, please refer to part 3 of this review. +, direct stimulatory effect; -, direct inhibitory effect; 0, no direct effect; AgRP, agouti related peptide; CART, cocaine and amphetamine regulated transcript; CCK, cholescystokinin; HK2, hemokinin 2; NKA, neurokinin A; NKB, neurokinin B; NMB, neuromedin B; NPY, neuropeptide Y; POMC, proopiomelanocortin; PYY, peptide YY; SP, substance P.

The control of food intake involves two major populations of ARC hypothalamic neurons, in mammals as well as in other vertebrates: neurons producing neuropeptide Y (NPY) and agouti-related peptide (AgRP), which are orexigenic (appetite stimulator) peptides, and neurons producing proopiomelanocortin (POMC) and cocaine-and-amphetamine-regulated transcript (CART), which are anorexigenic (appetite inhibitor) peptides [**Figure 3**; for reviews (12, 252–256)]. These hypothalamic neurons integrate information from peripheral hormones such as leptin, an anorexigenic hormone produced by adipose tissue in mammals and by liver in teleosts, and ghrelin, an orexigenic stomachal hormone [**Figure 3**; for reviews (12, 252–256)].

As gut peptides, tachykinins also have a potential direct role on the GIT in vertebrates. A well-described one is the stimulation of its motility, the first necessary step of food digestion after its intake [for review (257)].

### 4.1 Mammals

#### 4.1.1 Energy state and the tachykinin system

TAC3 neurons, mainly as part of KNDy neurons, have been involved in the regulation of both negative and positive energy balance. For example, they mediate the anorexigenic effect of estradiol in young female rats, as selective ablation of KNDy neurons suppresses the post-ovariectomy weight gain (258). In addition, many studies report the regulation of the tachykinin system by a change of energy status. Fasting and caloric restriction (CR) induce a decrease in hypothalamic ARC *tac3* and/or *tacr3* expression in rodents [pubertal female rat (259); OVX female mice (260); adult female rat (261)]. Nevertheless, in adult male mice, fast increases hypothalamic ARC *tac3* and *tacr3* (171) while CR and fast (261, 262) have no effect in adult female rats. In sheep, chronic food restriction downregulates *kiss* and *tac3* mRNA levels [castrated male sheep (263); OVX ewe lambs (264); for review (265)]. Feeding a high-fat diet does not change ARC mRNA levels for *tac3* in pubertal female mice (266) while it has a stimulatory effect in pubertal female rats (267). All these data suggest differential regulation of the tachykinin system (and of its involvement in the control of metabolism) according to species, sexual maturation stage, and/or degree of negative energy balance.

#### 4.1.2 Effects of tachykinins on food intake and GIT motility

Abundant distribution of TAC1, TAC2, and TAC3 receptors is found in the hypothalamic nuclei involved in the control of food intake such as ARC, paraventricular nucleus (PVN), and lateral hypothalamus (LHA) [for reviews (4, 97, 106)]. The full tachykinin system is also detected in the neurons and nerve fibers of the mammalian gut with remarkable diversity between species [for reviews (268–270)]. These distributions point out toward a potential involvement of TAC peptides in the control of feeding and GIT motility.

Administration of NPK to food-deprived rats for 24 h delays the onset of (re)feeding and decreases the cumulative food intake [(271); for review (272)]. Achapu and coworkers show that the inhibition of food intake induced by centrally injected NPK may be due to the intense grooming induced by the injection (273). Similarly, icv injection of SP to food-deprived male rats suppresses food intake, but an increase of locomotor activity is also observed (274). The fact that icv injections of NKA induce an increase in *pomc* mRNA levels in the rat ARC (275) also argues toward such anorexigenic action of tachykinins in mammals or at least in rodents. However, later, Karagiannides and collaborators consider SP as a novel antiobesity target after showing that the blockade of SP signaling by mean of an TACR1 antagonist leads to a decrease of food intake and body weight in two obese mouse models, an HFD-induced one and a leptin-deficient (*ob*/*ob*) one (276). They also report that peripheral injection of SP increases food intake and induces upregulation of hypothalamic *npy* as well as downregulation of *pomc* and mRNA levels (276). In male rats, ghrelin negatively regulates the *tac1* gene in the hypothalamus and acute icv injection of NPK and NPγ (but not SP nor NKA) reduces food intake (277). In addition, in male mice, the hyperphagic effect of peripheral injection of ghrelin disappears in *tac1*KO animals, suggesting the *tac1* requirement in the control of food intake by ghrelin in rodents (277). The first study using *tac1*-null mice does not show any difference in body size compared with controls (276), while a more recent one reports that these animals have a significantly lower body weight during adulthood and also show increased hypothalamic *pomc* expression and reduced food intake (278). All these data indicate that, in rodents, TAC1 peptides may function as either endogenous anorexigenic or orexigenic peptides.

The capacity of SP, NKA, and NKB to induce intestine contraction was one of the actions that led to their discoveries (refer to part 1.1. of this review). Their action on motility is observed in all parts of the gut through the tachykinin receptors and has been previously reviewed (279, 280).

### 4.2 Teleosts

#### 4.2.1 Energy state and the tachykinin system

In goldfish, a short-term postprandial increase in *tac1* mRNA levels (γ-PPT in the article) has been reported in both the hypothalamus and the olfactory bulbs (281). In zebrafish, fasting increases the brain expression of *tac3* in females (282). In grass carp, food intake can significantly induce hypothalamic *tac3a* and *tac3b* mRNA expression (222). Thus, in teleosts, change in energy state may have positive and negative effects on the TAC system.

#### 4.2.2 Effects of tachykinins on food intake and GIT motility

Immunohistochemical studies report high concentrations of tachykinins and their receptors in teleost hypothalamic areas involved in the control of food intake [SP in goldfish (283); SP in sea bass (152); carassin in goldfish (284); TACR1 and TACR3 in electric fish *Apteronotus leptorhynchus* (285)]. In goldfish, the *tac1* mRNA (γ-PPT in the article) encoding SP, carassin, and NKA presents a higher expression in olfactory bulbs and hypothalamus, while being present throughout the brain (58, 281). More recently, RT-PCR, qPCR, and ISH data confirmed the expression in the hypothalamus of the different *tac* and *tacr* genes [zebrafish (26, 71); tilapia (72); grass carp (61, 62, 75, 89); goldfish (73, 88); orange-spotted grouper (77); spotted sea bass (78); tongue sole (79)].

While tachykinins and their receptors are expressed in the teleost hypothalamus and its nuclei involved in the control of food intake, to our knowledge, no data have yet shown their direct effect on the expression of neuropeptides such as *pomc*, *npy*, or *agrp* at the brain level. Due to the direct innervation of pituitary cells by hypophysiotropic neurons in teleosts, Hu and colleagues demonstrated a high expression, in the brain and pituitary, of neuropeptides involved in the regulation of feeding (221). They subsequently reported that TAC3 and TAC4 peptides could change the expression of some of these genes, using transcriptomic analysis of TAC peptide effects on grass carp pituitary cells (62, 222). NKB can induce *in vitro* the expression levels of urotensin 1 (UTS1), cocaine-and-amphetamine-regulated transcript 2 precursor (CART2), proopiomelanocortin b (pomcb), and neuromedin B1 (NMB1) mRNA, all four anorexigenic peptides, an effect that is also reported *in vivo* after ip injection (222). HK2, a peptide encoded by the *tac4* gene in grass carp, upregulates CART2, CART3, and CART5, peptide YY2 (PYY2), UTS1, and NMB1 expression, while downregulating type 2 neuropeptide Y receptor (NPY2R) expression (62). Thus, NKB and HK2 inhibit the expression of the orexigenic pathway (such as NPY one) and stimulate anorexigenic peptides, playing roles of satiety factors (**Figure 3**). In grass carp, TACR3b mediates this role, while TACR3a modulates NKB action on reproduction (222), indicating a typical case of subfunctionalization where paralogs share initial pleiotropic functions. Tachykinins have also been involved in live prey food preference in hybrid *Siniperca chuatsi* × *Siniperca scherzeri* mandarin fish as *tac 1* expression is higher in feeders compared with non-feeders in this species (286).

Tachykinins and their receptors are also expressed in the stomach and intestine of many teleosts, suggesting potential autocrine or paracrine actions [zebrafish (26, 71); tilapia (72); goldfish (73, 88); grass carp (61, 75, 211); orange-spotted grouper (77); spotted sea bass (78); tongue sole (79)].

Peripheral action with direct contraction of the smooth gut muscle has also been described in teleosts [for reviews (10, 287)]. Substance P stimulates the motility of isolated intestine or stomach in a variety of fish [*Pleuronectes platessa*, *Labrus bergylta*, *Gadus* species, *Lophius* species, *Anguilla* species (55); rainbow trout (288, 289); cod *Gadus morhua* (290, 291); common carp *Cyprinus carpio* (292); bichir *Polypterus senegalensis* (293)]. In many of these species, it is demonstrated that the effect of SP is in part direct (cotreatment with tetrodotoxin, a sodium channel blocker) and in part *via* stimulation of cholinergic and serotonergic neurons (cotreatment with cholinergic or serotonergic antagonists, atropine and methysergide) [**Figure 3**, common carp (294):; rainbow trout (288, 289, 293)]. NKA is also able to stimulate the motility of isolated trout intestinal muscle and the vascularly perfused trout stomach, but with less efficiency than SP (289). This stimulatory control of gut motility by the tachykinin system takes place at an early stage in development as NKA modulates zebrafish larval gut before or around the time for the onset of feeding (295). More recently, an increase in the expression of *tac1* has been detected by RNA-seq in the giant grouper *Epinephelus lanceolatus* at the onset of feeding (296). Using *in vitro* stomach and intestine incubation assays in the sea bass, Zhang and collaborators showed that NKB peptides may modulate the expression of hormones (78) (**Figure 3**), known to have stimulatory activity on GIT motility in vertebrates, such as motilin and ghrelin [for review (297)]. In the stomach, NKBa-13 and NKBb-13 stimulate gastrin mRNA levels, while NKB-10 peptides have no effect (78). NKBb-13 can stimulate stomachal motilin and ghrelin expression, while the other three NKB peptides have no effect (78). In the intestine, NKBa-13, NKBa-10, and NKBb-13 stimulate cholecystokinin mRNA levels, while NKBb-10 has no effect (78). Only NKBb-10 can stimulate intestinal gastrin expression and NKBa-10 motilin expression. None of the four NKB peptides can change ghrelin mRNA levels in the intestine (78).

## 5 Conclusions and perspectives

Cumulating evidence places the tachykinin system with not only NKB (TAC3) but also other tachykinin peptides, SP (TAC1) and NKA (TAC1), as major stimulatory actors in the control of reproductive function, in mammals. In teleosts, the two TAC3 peptides NKB and NKBRP and their 3R duplicates can have various effects (stimulatory, inhibitory, or none) mainly according to the species, the maturity stage, and the peptide tested. These sometimes opposite effects of TAC3 peptides on reproductive genes among teleost species are also reported for other neuropeptides involved in the control of the HPG axis, such as kisspeptin and gonadotropin-inhibitory factor (GnIH) (for review: 9). One may take into consideration the variety of reproductive strategies and life cycles among these more than 25,000 different species to try to find explanations, as well as the physiological compensations between neuropeptides that are likely to exist in teleosts, perhaps due to the anatomical direct innervation of pituitary cells and the existence of various 3R paralogs. Concerning potential involvement of TAC1 and TAC4 peptides in teleost reproduction, too few data are available to draw any conclusion. Past studies in mammals and recent ones in teleosts suggest that the tachykinin system may also be involved in the regulation of food intake and metabolism. Even if more studies are still needed, especially concerning the role of TAC4 peptides, it looks like the control of food intake may be taken over by TAC1 peptides in mammals but also by TAC3 and 4 peptides in teleosts. Tachykinins and their receptors thus seem to be part of networks linking metabolism and reproduction and involving central and peripheral hormones, such as kisspeptin, leptin, and ghrelin.

Due to the multiple whole-genome duplication events that occurred in vertebrates, phenomena of divergence and subfunctionalization or neofunctionalization of the ancestral functions are expected and observed, especially in teleosts. Therefore, an analysis of the tachykinin system is recommended for each organism of interest in order to obtain a clear view of the function of this family of peptides and receptors according to vertebrate species.

Future studies should aim at stating whether or not KNDy neurons exist in some teleost species. Too few species have been considered so far. Surprisingly, up to now, no study has yet investigated the possible direct effects of TAC3 peptides on pituitary LH and FSH cells in mammals; this should be performed in the future using primary cultures of pituitary cells from different mammalian species. It would also be interesting to study whether TAC peptides could directly modify the expression of central actors involved in the control of food intake, such as *pomc* and *npy*, in both mammals and teleosts. Future directions on the study of the tachykinin system should also include investigations on Mas-related GPCRs (Mrgprs), as TACR1 antagonists have off-target activity on them (298) and substance P recruits these receptors in immune cells to release cytokine contributing to inflammatory pain in mice (299, 300). For example, characterizing Mrgprs in teleosts and knowing whether they are present in the HPG tissues will help to decipher their potential involvement in the reproductive role of the tachykinin system.

## Author contributions

All authors contributed to the article and approved the submitted version.
